# Improved drought tolerance of EMS mutagenized Alfalfa (*Medicago sativa* L.) mutants by in vitro screening at germination stage

**DOI:** 10.1038/s41598-022-16294-0

**Published:** 2022-07-26

**Authors:** Iskender Tiryaki, Ugur Sari, Selcuk Cetin, Okan Acar

**Affiliations:** 1 Department of Agricultural Biotechnology, Faculty of Agriculture, Canakkale Onsekiz Mart University, Terzioglu Campus, 17000 Canakkale, Turkey; 2 Department of Biology, Faculty of Arts and Sciences, Canakkale Onsekiz Mart University, 17100 Canakkale, Turkey

**Keywords:** Molecular biology, Plant sciences

## Abstract

The objectives of this study were to determine drought tolerant novel mutant of alfalfa **(***Medicago sativa* L.**)** genotypes by screening EMS mutagenized 340675 M_3_ seeds at germination stages in the presence of osmotic stress of 35% PEG_6000_. Root growth assay provided several drought tolerant candidate mutants. Of those, 4 mutants were further evaluated at water deficit conditions applied for 24 days after the first cutting at flowering bud stage. The results revealed that mutants determined as drought tolerant at germination stage were also tolerant to water deficit conditions. Protein content and superoxide dismutase values were found to be higher in all mutants than controls. Ascorbate peroxides, glutton reductase and lipid peroxidase values varied based on the mutant genotype and duration of drought stress. Drought stress significantly changed transcriptional levels of *MtP5CS*, *MtDehyd*, *MseIF*-2, *MtRD2* and *MsNAC* genes. These results indicated that in vitro screening of alfalfa mutant seeds for osmatic tolerance at germination and early seedling growth stages was successfully able to determine the drought tolerant alfalfa mutants which were also tolerant to water deficit conditions after the first cutting at flowering bud stage.

## Introduction

Global warming is threatening today’s agriculture production by limiting the irrigation water needed for plant production^1^. Therefore, the development of new plant varieties that can better tolerate drought stress conditions and minimize yield losses has a strategic importance in terms of ensuring the food security of future generations. However, the complex structure of the drought stress mechanism is one of the most important reasons for the slow progress of breeding studies^2^. Drought tolerance shows a quantitative inheritance controlled by several genes^3^. The epistatic and pleiotropic relationships between these genes also make it extremely difficult to put into practice for breeding studies^3^. In addition, factors such as development periods of a plant, duration and severity of stresses are also important determinants for evaluating the extent of quantitative inheritance of drought stress^4^.

Drought tolerant plants have ability to perform their normal functions even at low water potential conditions^5^. Plants use several strategies under water-deficit conditions to minimize deleterious effects of drought stress at physiological, morphological, and transcriptional levels. Plants also use an escape strategy which is defined as the ability of plants to maintain high water potential under drought condition. This strategy is generally provided by some agro-morphological changes in plants, such as reducing the leaf area, reducing the number and conductivity of stomata, forming dense root systems and increasing the root/stem ratio^6^.

Alfalfa (*Medicago sativa* L.) is an essential forage crop and has a significant economic importance worldwide due to its invaluable contribution to sustainable agriculture and husbandry in various ways^7^ including high hay yield, outstanding nutritional quality, and nitrogen-fixing ability^8^. Varieties cultivated within the alfalfa species are autotetraploid with almost identical genomes^9^. The alfalfa is generally shown as drought tolerant due to its deep root system which is generally pronounced in the few years following the planting^10^. However, along with germination and early seedling growth, regrowth stage of alfalfa right after cutting in the planting year are very vulnerable to drought stress. In addition, alfalfa needs much more irrigation water compared to other cultivated plants, especially in arid areas, since it is harvested several times and provides the highest-yielding forage in a growing season.

Although partial success has been made in the development of new alfalfa varieties which tolerate various abiotic stresses, including drought^11^, very limited progresses have been made in comparison to other important crop species^12^. In addition, identifying tolerant plants against to drought stress by direct selection mainly depends on both the genetic variation of the material used and the success of the screening methods. It appears to be very difficult to develop new drought tolerant alfalfa genotypes by using crossbreeding method and by screening existing a narrow genetic base of alfalfa^13^. Therefore, we have used ethyl methane sulfonate (EMS) mutagenesis to create a novel genetic variation. Screening M_3_ mutant seeds at germination stage under in vitro conditions gave several drought tolerant candidates. Four of them were also re-evaluated at the physiological, morphological, and transcriptional levels under water-deficit conditions applied for 24 days after the first cutting at flower bud stage which was also considered as another critical stage to determine regrowth performance of alfalfa under both irrigated and unirrigated growth conditions. Drought stress responses of mutants were compared with irrigated and unirrigated control plants.

## Results

### M_3_ mutants showed a better regrowth performance under water-deficit conditions

**Figure 1:**
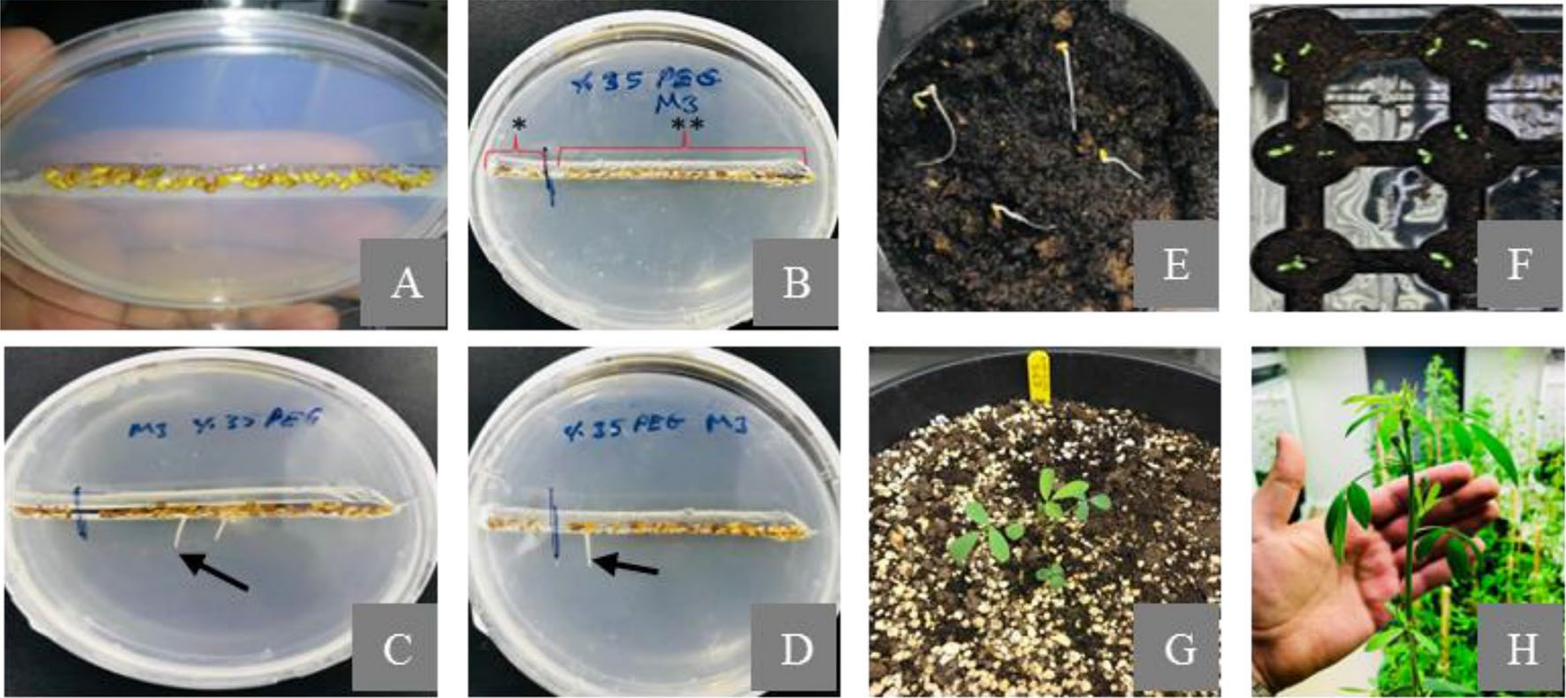
Screening mutant M_3_ seeds and developed seedlings. (**A**) Close view of planted seeds on the raw of MS media containing 35% PEG_6000_. (**B**) *, unmutated control seeds; **, mutant M_3_ seeds. (**C**–**D**) Candidate mutant seedlings from screening. Arrows show radicle elongations. (**E**–**F**) Rescued seedlings from MS media and planted into viols. (**G**–**H**) Seedlings planted into plastic pot and plant developed at flowering bud stage.

Screening of 340675 M_3_ seeds at germination and early seedling growth stages in the presence of osmotic stress of 35% PEG_6000_ resulted several drought tolerant candidates (Fig. 1). Of those, 4 mutants were further tested for re-growth performances under water deficit conditions applied for 24 days after the first cutting at flowering bud stage and the results were compared with both irrigated (Z1) and unirrigated control plants (Z2) (Table 1).

**Table 1:** Agro-morphological parameters determined on the 18th and 24th days of drought stress applied after first cutting at budding stage.

Mutant code	Plant height (cm)	Main stem thickness (mm)	Natural plant height (cm)	The number of lateral branches per plant	The number of leaves per plant	The middle leaflet length (mm)	The middle leaflet width (mm)	Plant canopy temperature (°C)	Petal colour, ƒ	The number of seeds per pod
**On the 18th day of drought stress**										
Z1	42.1	2.3	31.3	2	53	27.7abc	18.0ab	20.5b	3	0.80bc
Z2	38.0	0.8	19.7	0	27	22.7c	14.0bcd	20.3b	3	1.75ab
X6	36.3	2.3	20.6	2	49	30.0ab	20.0a	21.5a	4	2.00a
Y20	39.5	2.6	12.7	9	189	31.0a	16.5abc	19.5c	1	1.0abc
Y30	32.5	2.5	12.4	8	78	16.2d	12.5cd	19.6c	3	0.6ab
Y35	45.7	2.1	20.1	4	79	25.0bc	11.0d	20.4b	4	0.00c
LSD_0.05_						5.4	4.6	0.5		1.19
**On the 24th day of drought stress**										
Z1	75.3	3.1	53.0	8	370	25.2a	11.0ab	20.5a		
Z2	52.0	0.7	26.0	5	96	11.5c	6.5c	20.5a		
X6	58.3	2.7	50.5	9	126	25.5a	13.5a	20.4a		
Y20	36.0	2.1	22.4	4	87	23.2ab	11.2ab	19.1b		
Y30	54.6	2.8	16.8	11	63	20.0b	10.2b	20.5a		
Y35	59.6	2.4	26.9	7	42	26.0a	12.0ab	20.4a		
LSD_0.05_						4.7	2.8	0.4		
						*n* = 4	*n* = 4	*n* = 3		*n* = 5

^ƒ^1, light pink; 2, pink; 3, light purple; 4, purple. Different letters indicate significant differences at *P* < 0.05*.*

The results of agro-morphological parameters revealed important variations not only within the M_3_ mutants, but they also showed significant differences compared to the control plants (Table 1). The plant height of the mutants on the 18th day of drought stress ranged from 36.3 cm to 45.7 cm while Z1 and Z2 controls had 42.1 cm and 38 cm plant height, respectively (Table 1). When drought stress was extended to 24 days, all mutants with one exception (mutant Y20) gave a longer plant height than the control Z2 (52.0 cm). The main stem has generally thickened as drought stress extended from the 18th to 24th days with exception of mutant Y20 and the control Z2 (Table 1). The effects of drought stress on natural plant height of mutants varied based on drought duration. The number of lateral branches ranged from 2 to 9, and from 4 to 11 per plant on the 18th and 24th days of drought stress, respectively. Except mutant Y20, span of drought stress increased the number of lateral branches in all mutants as well as in the control plants (Table 1). The number of leaves per plant increased on the controls and the mutant X6 plant while decreased number of leaves per plant was determined on the mutants Y20, Y25 and Y35 when the drought stress was extended from 18 to 24th days (Table 1). The drought stress reduced the middle leaflet length and width in all mutants and the controls on the days of 18th and 24th of drought stress (Table 1). However, the reduction rate was less on the mutants than the control Z2 plant (Table 1). The plant canopy temperatures were found to be significantly different between the controls and the mutants on the days of 18th and 24th of drought stress (Table 1). The mutants Y20 and Y30 had less canopy temperature than both controls on the 18th day of drought stress while canopy temperature was significantly lower on the mutant Y20 than the other mutants on the day of 24th of drought stress (Table 1). The highest number of seeds per pod (2 seeds/pod) and the highest seed yield per plant (1.34 g/plant) were obtained from the mutant X6 while mutant Y35 did not give any seed (Table 1). The flower colour of the mutants varied from pink to purple while the controls had pink colour only (Table 1).

### The effects of water deficit conditions on physiological parameters of mutants

**Figure 2:**
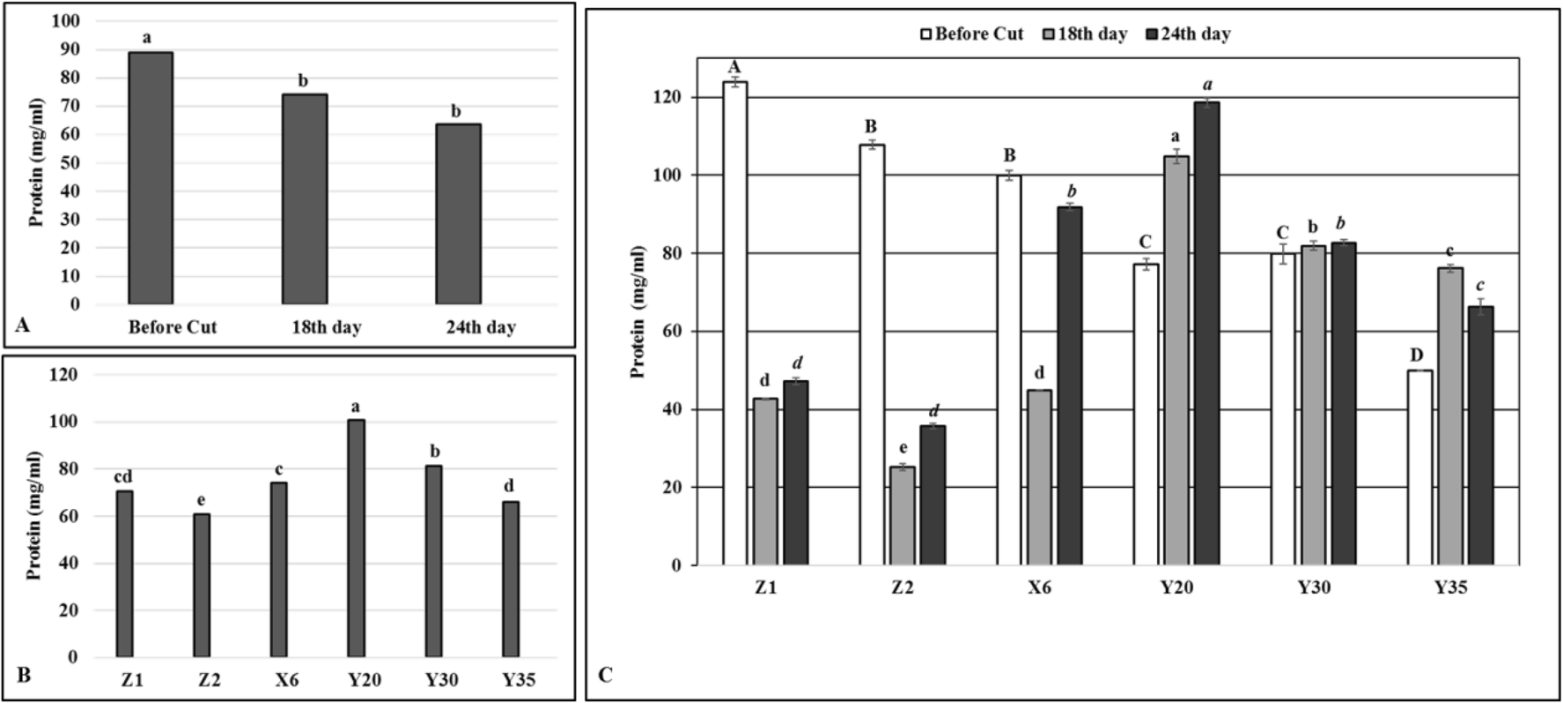
Protein contents. (**A**) Overall effects of drought stress to mutants and control plants at given time intervals, *n* = 18. (**B**) Overall protein contents of mutant and control plants, *n* = 9. (**C**) Effects of drought stress on protein contents of mutants and control plants at given time intervals, error bars indicate standard deviation, *n* = 3. Different letters indicate significant differences at *P* < 0.05. Capital, small or small italic letters indicate statistical analysis carried on given time intervals.

Drought stress significantly reduced protein contents of alfalfa (*p* < 0.001) (Fig. 2A). However, overall protein content in mutants Y20 and Y30 were found to be higher than both controls (Z1 and Z2) plants while the mutants X6 and Y35 had the same level of protein contents with irrigated control (Z1) (Fig. 2B). Within mutants, the mutant Y20 gave the highest overall protein content (100.68 mg/ml) while mutant Y35 had the lowest (66.04 mg/ml) considered all time intervals (Fig. 2B). The protein levels of mutant Y20 significantly increased as drought stress span while it was stable in the mutant Y30 at all time intervals tested (Fig. 2C). The highest protein levels (104.9 mg/ml and 118.60 mg/ml) were obtained from the mutant Y20 while the control Z2 had 25.30 mg/ml and 35.75 mg/ml on the 18th and 24th days of drought stress, respectively (Fig. 2C).

**Figure 3:**
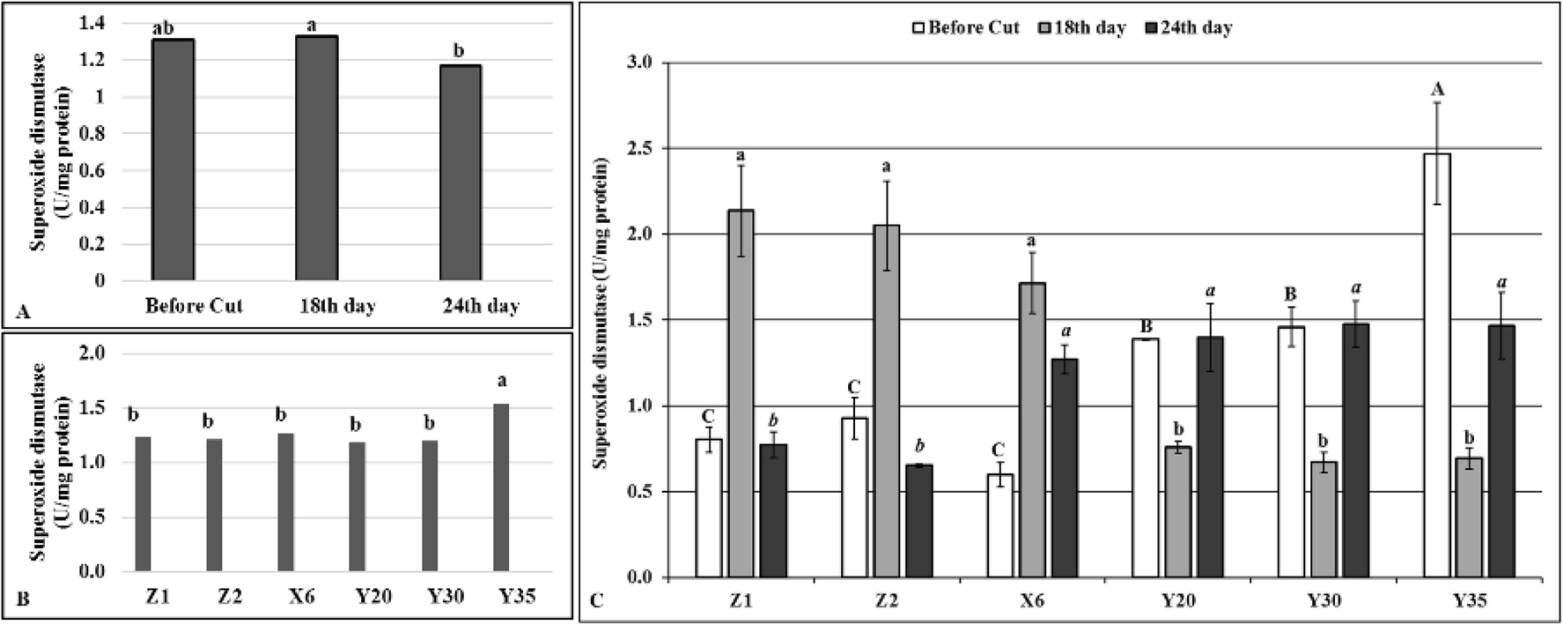
Superoxide dismutase (SOD) isoenzyme levels. (**A**) Overall effects of drought stress to mutants and control plants at given time intervals, *n* = 18. (**B**) Overall SOD levels of mutant and control plants, *n* = 9. (**C**) Effects of drought stress on SOD levels of mutant and control plants at given time intervals, error bars indicate standard deviation, *n* = 3. Different letters indicate significant differences at *P* < 0.05. Capital, small or small italic letters indicate statistical analysis carried on given time intervals.

The drought stress significantly decreased SOD isoenzyme levels (Fig. 3A) and the mutant Y35 had the highest SOD level (1.54 U/mg protein) (Fig. 3B). Eighteen day of drought stress significantly increased SOD levels in both controls and mutant X6 plants although the SOD levels significantly decreased to a level observed before cutting stage in the other mutants (Fig. 3C). In contrast to the controls and mutant X6, the SOD enzyme levels significantly elevated on the 24th of drought stress on the mutants (Fig. 3C).

**Figure 4:**
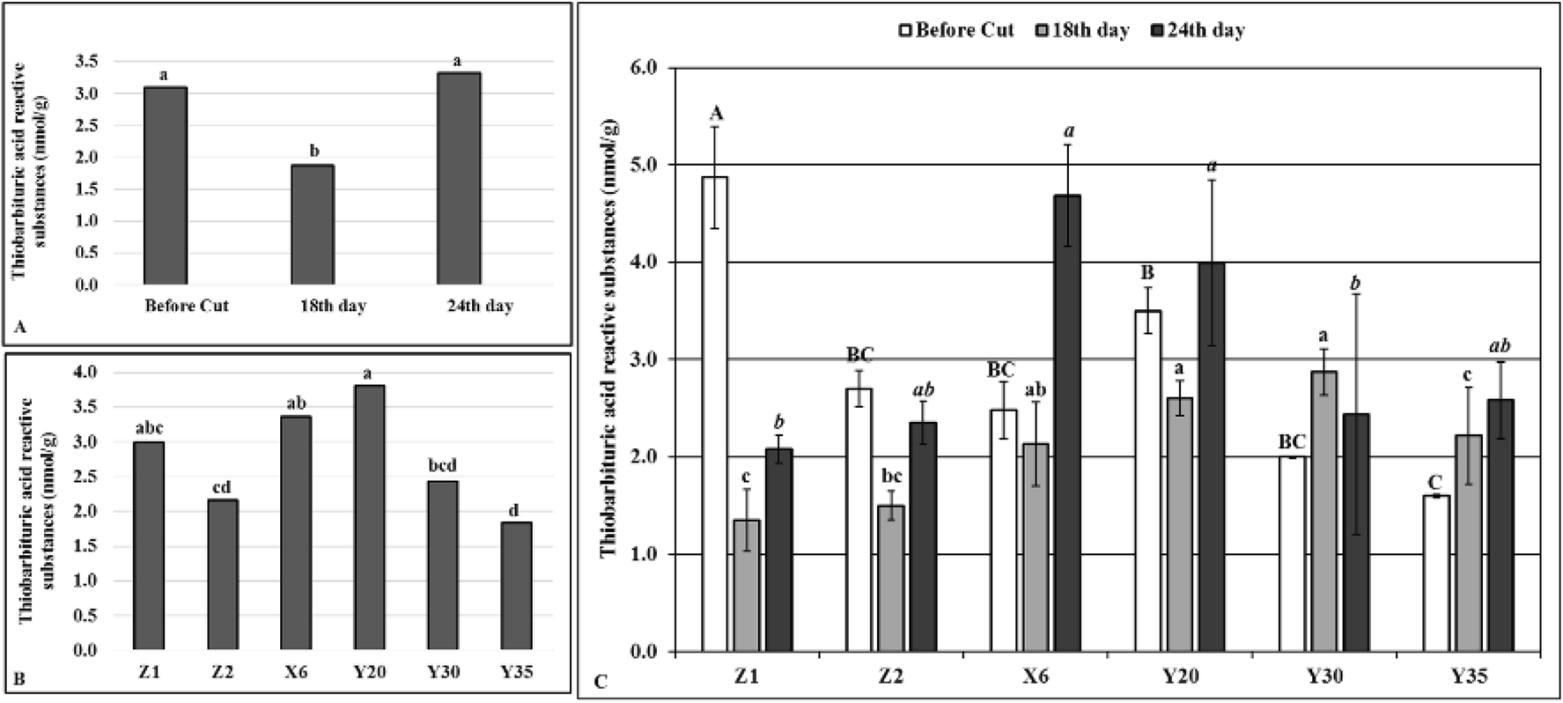
Thiobarbituric acid reactive substances (TBARS) levels. (**A**) Overall effects of drought stress to mutants and control plants at given time intervals, *n* = 18. (**B**) Overall TBARS levels of mutant and control plants, *n* = 9. (**C**) Effects of drought stress on TBARS levels of mutant and control plants at given time intervals, error bars indicate standard deviation, *n* = 3. Different letters indicate significant differences at *P* < 0.05. Capital, small or small italic letters indicate statistical analysis carried on given time intervals.

The first period of drought stress (18 days) decreased the TBARS levels (Fig. 4A) while no significant differences were detected between the time periods of before cutting and on the 24th day of drought stress (Fig. 4A). The highest TBARS levels (3.80 and 3.36 nmol/g protein) were obtained from the mutants Y20 and X6, respectively, while the mutant Y35 had the lowest (1.83 nmol/g protein) (Fig. 4B). The mutant Y35 increased TBARS level as drought stress extended from 18 to 24^th^ days while the mutant Y30 decreased it (Fig. 4C). In general, a similar pattern of TBARS levels were detected on the controls and mutants Y20 and X6 on given time intervals of drought stress in comparison to TBARS levels of before cutting stage (Fig. 4C).

**Figure 5:**
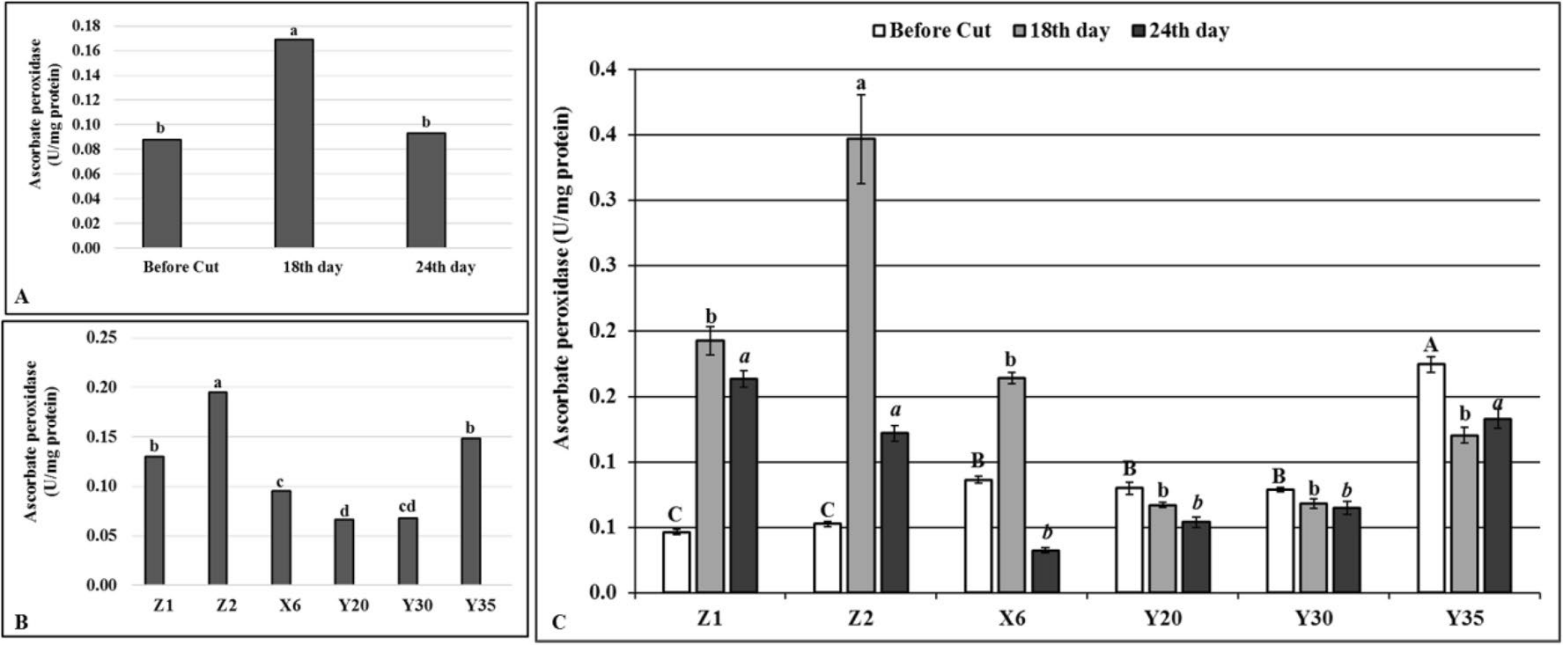
Ascorbate peroxidase (APX) enzyme activity. (**A**) Overall effects of drought stress to mutants and control plants at given time intervals, *n* = 18. (**B**) Overall APX levels of mutant and control plants, *n* = 9. (**C**) Effects of drought stress on APX levels of mutant and control plants at given time intervals, error bars indicate standard deviation, *n* = 3. Different letters indicate significant differences at *P* < 0.05. Capital, small or small italic letters indicate statistical analysis carried on given time intervals.

The highest (0.17 U/mg protein) APX enzyme activity was determined on the 18th day of drought stress in comparison to the other time intervals tested (Fig. 5A). The APX activity was the highest (0.19 U/mg protein) on the control Z2 while no significant differences were detected between the mutant Y35 (0.148 U/mg protein) and the control Z1 (0.130 U/mg protein) considered overall time periods (Fig. 5B). The mutant Y35 had the highest overall APX level in comparison to the other mutants (Fig. 5B). The APX activity was the highest on the mutant Y35 while both controls had the lowest APX activity on the time of before cutting (Fig. 5C). The APX enzyme activity significantly increased on the 18^th^ day of drought stress on the controls and the mutant X6 compared to the levels determined before cutting while statistically the same APX enzyme activity levels were determined on irrigated control (Z1) and mutants on the 18th day of drought stress (Fig. 5C). Except the mutant Y35, reduced APX enzyme activity levels were determined on the control and mutant plants on the 24th day of drought stress compared to the 18th day of drought stress although the mutant Y35 had the same level of APX enzyme activity with both controls (Fig. 5C).

**Figure 6:**
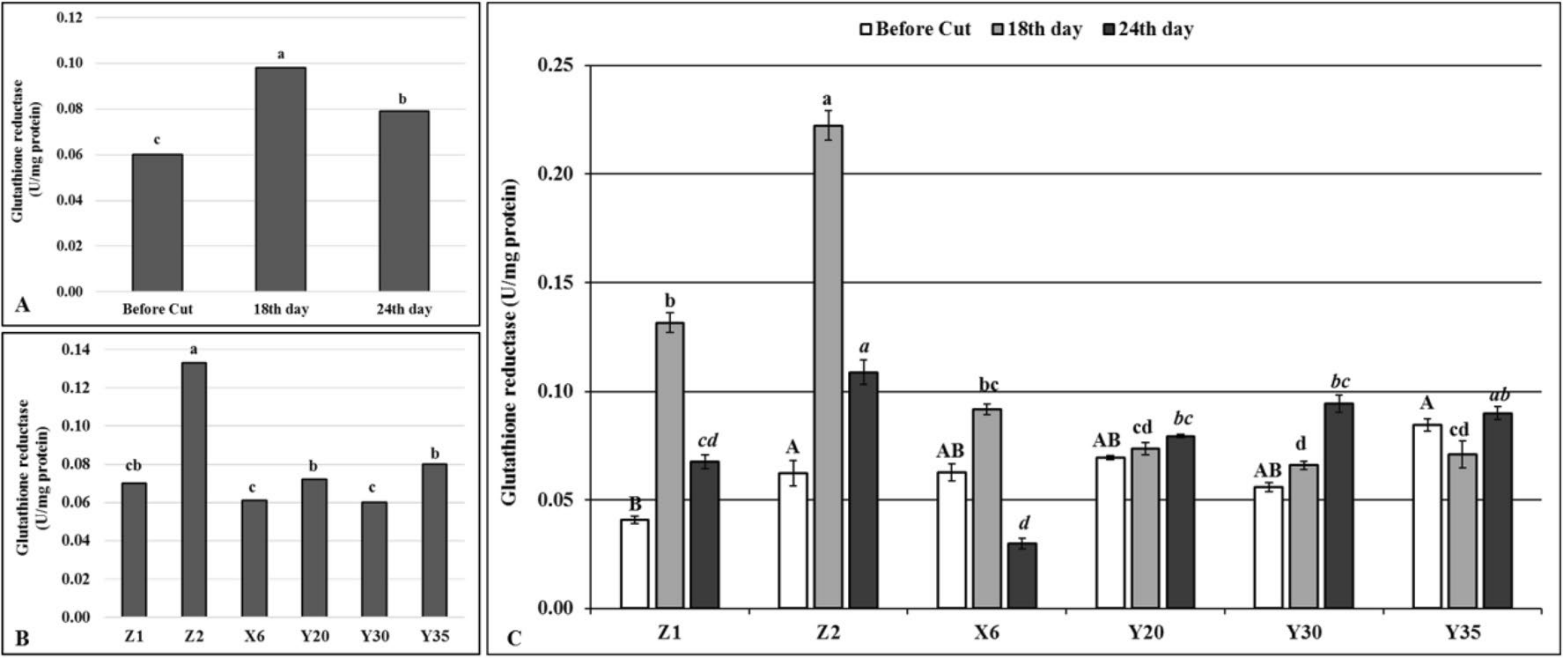
Glutathione reductase (GR) enzyme activity. (**A**) Overall effects of drought stress to mutants and control plants at given time intervals, *n* = 18. (**B**) Overall GR levels of mutant and control plants, *n* = 9. (**C**) Effects of drought stress on GR levels of mutant and control plants at given time intervals, error bars indicate standard deviation, *n* = 3. Different letters indicate significant differences at *P* < 0.05. Capital, small or small italic letters indicate statistical analysis carried on given time intervals.

The GR levels significantly increased during the drought stress in comparison to before cutting (Fig. 6A). Although a significant decrease was observed at the end of drought stress, overall GR level was significantly higher than before cutting stage (Fig. 6A). The highest overall GR level (0.098 (U/mg protein) was obtained from the control Z2 while no significant differences were determined on the mutants and control Z1 (Fig. 6B). Significant differences were determined on the GR levels of controls and mutant plants for all given time periods (Fig. 6C). The irrigated control (Z1) gave the lowest GR level on the before cutting stage while statistically the same GR levels were determined on the control Z2 and mutants (Fig. 6C). Eighteenth day of drought stress significantly increased GR levels in both control and mutant plants, except mutant Y35, although GR amount was relatively lower on the mutants than control plants (Fig. 6C). Except mutant X6, the mutants showed an increased level of GR on the 24th day of drought stress while a reduced GR content was determined on the controls compared to the 18th day of drought stress (Fig. 6C). A similar pattern of GR content was determined on the mutant X6 and control plants on given time periods (Fig. 6C).

### Drought stress changed expression profiles of drought-specific genes in mutants

**Figure 7:**
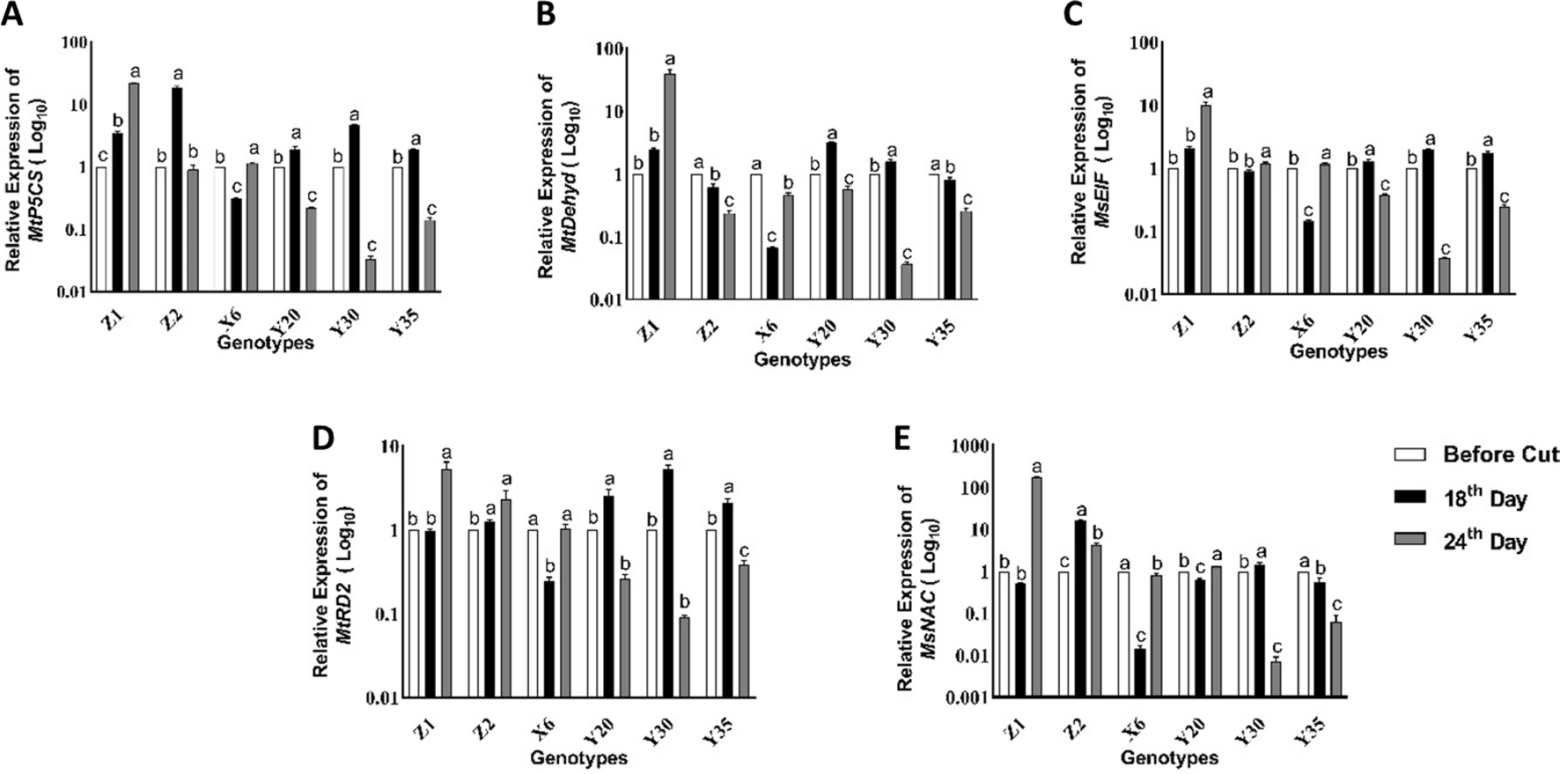
Relative expression levels of drought responsive genes at given time intervals (**A**–**E**) Changes in expression of *MtP5CS* (*Medicago truncatula pyrroline-5-carboxylate synthetase*) (**A**), *MtDehyd* (*Medicago truncatula dehydrin*) (**B**), *MseIF-2* (*Medicago sativa* eukaryotic translation initiation factor 2) (**C**), *MtRD2* (*Medicago truncatula Response to Desiccation 2*) (**D**) and *MsNAC* (*Medicago sativa NAC*; NAM, ATAF, and CUC2) (**E**) genes in leaf tissues of alfalfa mutant plants. *Ms18s rRNA* (*18S ribosomal RNA*) was used as the reference gene. The results shown are means ± standart error, *n* = 3. Different letters indicate significant differences at *P* < 0.05*.*

The *MtP5CS* gene expression showed great variation, with increases of 0.31-fold (X6) to 4.71-fold (Y30) on 18th day of drought stress although the expression levels declined to 0.03 (mutant Y30) and 1.14-fold (X6) on the 24th day of drought stress (Fig. 7A). On the other hand, the expression levels of *MtP5CS* gene declined on control Z2 from 18.6-fold to 0.9-fold when drought stress was extended from 18 to 24 days while the control Z1 increased the expression level of the same gene from 3.49-fold to 21.75-fold on the same time intervals (Fig. 7A). Except the mutant X6, similar expression patterns were observed on the mutants in response to drought stress while control Z1 showed an elevated *MtP5CS* gene expression as drought stress span from 18 to 24 days (Fig. 7A).

The regulation of *MtDehyd,* also known as *LEA* (*late-embryogenesis abundant*) gene, showed great changes in response to drought stress applied after the first cutting at bud stage and regulation levels of related gene were found to be genotype dependent (Fig. 7B). The expressions of *MtDehyd* gene on the 18th day of drought stress varied from 0.07-fold (X6) to 3.12-fold (Y20) compared to the reference gene (Fig. 7B). The *MtDehyd* gene expression significantly decreased on the mutants X6, Y35 and control Z2 plants on the 18th day of drought stress while increased expression levels of the same gene were observed on the mutants Y20, Y230 and control Z1 plants at the same time intervals compared to before cutting stage (Fig. 7B). The *MtDehyd* gene expression was up-regulated on the control Z1 (from 2.42 to 39.23-fold) and mutant X6 (from 0.07 to 0.45-fold) when drought stress was extended from 18 to 24 days (Fig. 7B).

Regulation of the *MseIF-2* gene expression varied depended on mutant genotype and duration of drought stress (Fig. 7C). For example, the mutants Y20, Y30 and Y35 showed 1.26-fold, 1.97-fold and 1.71-fold increase on the 18th day of drought stress while it has been significantly diminished to 0.38-fold, 0.04-fold and 0.25-fold changes on the 24th day of drought stress, respectively (Fig. 7C). On the other hand, the *MseIF-2* gene expression of the mutant X6 was declined on the 18th day of drought stress and then increased on the 24th day of drought stress compared to before cutting (Fig. 7C). The expression levels of *MseIF-2* gene significantly elevated on both control plants (Z1 and Z2) as drought stress extended from 18 to 24 days while significant decreases were observed on mutants with exception of mutant X6 (Fig. 7C).

Extending duration of drought stress from 18 to 24 days caused significant decline on the expression levels of *MtRD2* gene on the mutants Y20, Y30 and Y35 while it caused significant increases on both control plants (Z1 and Z2) (Fig. 7D). The highest (5.25-fold) and the lowest (0.09-fold) the *MtRD2* gene expression were determined on the mutant Y30 considered 18th and 24th days of drought stress, respectively (Fig. 7D).

The *MsNAC* gene expression showed a great variability and magnitude has changed based on the time and duration of drought stress in the controls and mutant plants (Fig. 7E). For instance, there was no significant difference on the control Z1 plant on the 18^th^ day of drought stress while 16.01-fold increase was determined on the control Z2 plant at the same time interval (Fig. 7E). The expression of *MsNAC* gene showed a significant decrease on the mutants X6 (100.4-fold), Y20 (0.63-fold) and Y35 (0.55-fold) although the mutant Y30 showed 1.44-fold increase on the 18th day of drought stress (Fig. 7E). A huge increase on *MsNAC* gene expression (177.32-fold) was determined on the irrigated control (Z1) plant while a great decline (100.70-fold decrease) was observed on the mutant Y30 on the 24th day of drought stress in comparison to reference gene (Fig. 7E).

## Discussion

Drought stress can cause a significant yield loss and magnitude can be vary depending on not only its intensity and severity, but also the developmental stages of plant^14^. Like in many other important crops species, seed germination stage of alfalfa is very vulnerable to drought stress^15^. Therefore, new alfalfa cultivars which tolerate drought stress better at germination stage are needed to minimize and to ensure production stability of alfalfa. Previous reports indicated that osmotic substances with high molecular weight such as PEG are one of the most popular approaches for screening of target genotypes at germination or other development stages of many plants including alfalfa^16^. A positive correlation between germination on PEG supplemented media and the whole plant behavior under water deficit conditions in the field was also reported^17^. However, the laboratory screening methods which simulate water deficiency and drought stress conditions should be reliable for determining of desirable genotypes of successful breeding programs^18^. Screening of 340675 M_3_ seeds in the presence of 35% PEG_600_ supplemented media under in vitro germination condition yielded several drought tolerant candidates which showed a visible and measurable radicle growth while no germination was observed on the control seeds under the same stress conditions, indicating that root growth assay and germination conditions used in the study were able to determine novel drought tolerant mutants which were able to accomplish cell division and enlargement as well as cell differentiation (Fig. 1). It is well known that PEG cannot pass through the cell wall due to its high molecular weight and are able to regulate water potential of embryonic cells via controlling poor water flow from xylem to the nearby cells and limits the process of cell growth mainly due to the loss of turgor^19^, resulting impaired cell elongation and inhibition of seed germination^20^. The germination variation in the simulated drought stress with PEG has also been reported for other important crops including alfalfa^21^, clover^16^ and wheat^22^.

Seed germination is initial but not only prerequisites for successful seedling establishment for drought tolerance since seedlings exposed to drought stress may not survive during recovery or retain some growth disorders in later growth stages^23^. In addition, the results of laboratory simulated water stress conditions at germination stage should be confirmed under real water deficit conditions at different plant growth stages^24^. Therefore, we have further tested 4 candidate mutants under water deficit conditions established for 24 days right after the first cutting at flower bud stage. The results of the current study revealed that the M_3_ mutants showed a great variation and deleterious effects of drought stress on agro-morphological parameters changed based on both mutant genotype and duration of the drought stress applied (Table 1). All mutants had better regrowth performances and tolerated drought stress conditions better than unirrigated control plant (Z2) on the given time intervals suggesting that decrease on photosynthesis and availability of photoassimilates under drought stress conditions limited in mutants compared to control plant (Table 1). Previous reports indicated that water stress reduced the number of leaves and leaf size and provided a lower biomass in alfalfa although the number of lateral branches increased particularly under severe drought conditions^25^ which agreed with the corresponding results of this study (Table 1). The main reasons for the reduced plant leaf area under drought stress were shown the decreased leaf turgor pressure, canopy temperature, and availability of photoassimilates due to decrease of photosynthetic rate under drought stress conditions^26^. The stomatal limitation was shown one of the main factors for decreased photosynthetic rate under mild drought condition although non-stomatal factors such as decrease photosynthesis were shown the main reason for the decline of the photosynthetic rate under severe drought conditions^26^.

Severe drought stress decrease hay yield and crude protein (CP) content and increase fiber which decrease the digestibility of the herbage in alfalfa although impact of drought stress on yield and composition of alfalfa could be vary based on alfalfa cultivar^27^. Previous report indicated that drought stress decreased the ratio of CP to the fraction of water-soluble carbohydrates which could reduce the N surplus in ruminates^27^. In contrast to protein contents of both irrigated (Z1) and unirrigated control (Z2) plants, drought stress increased protein contents of the mutants Y20 and Y30 while it did not change on the mutant Y30 (Fig. 2). These finding suggested that EMS have caused various type of point mutations in the alfalfa genome and protein biosynthesis pathway of the mutants differently regulated in response to drought stress compared to the controls. Since a high protein level is a crucial importance for animal feeding under drought stress conditions, the novel mutants determined in this study may be used as an important resource to develop new drought tolerant alfalfa varieties in breeding programs.

Drought stress initially causes formation of reactive oxygen species (ROS) which causes oxidative damages by preventing functions of lipids and proteins in the cells^28^. To prevent or to diminish the deleterious effects of drought stress, plants use either enzymatic or non-enzymatic antioxidant defense system^29^ although enzymatic defense system is generally considered as the most effective^30^. Antioxidant enzymes prevent naturally occurring ROS appeared as a result of the metabolic activities of the cells from damaging subcellular structures^28^. It is well known that the ROS production also increases in response to drought stress^28^ and antioxidant enzymes such as APX, GR and SOD play very important roles in detoxifying of ROS and protect cell from potential damage that may occur with increased ROS^31^. Drought tolerant alfalfa and some other legume plants become more tolerant to the negative effects of drought and salt stresses by increasing their antioxidant enzyme activities ^32^. The results of this study showed that the SOD, APX and GR contents of the mutants distinctly differ from unirrigated control (Z2) (Figs. 3, 5, 6). For instance, the SOD enzyme activities decreased on the 18th day of drought stress while the same enzyme activity increased on the 24th day of drought stress on the mutants Y20, Y30 and Y35 in comparison to controls and mutant X6 (Fig. 3). These findings suggested that a decreased SOD enzyme activity on the 18th day of drought stress in given mutants may not be directly related to drought stress per se but cutting effects at flowering bud stage (Fig. 3). It is also possible that the mutants might sense drought stress later in time than control plants and increased level of the SOD enzyme activity on the 24th day of drought stress indicates an improved drought tolerance on the mutants due to random point mutation of EMS mutagen (Fig. 3).

The TBARS is one of the most common parameters to detect lipid oxidation in response to stress^1^. The MDA is a split product of an endoperoxide of unsaturated fatty acids and reacts with thiobarbituric acid (TBA) forming TBARS^33^. Prolonged drought stress increased TBARS levels on the mutants X6 and Y20 while similar TBARS levels were determined on the mutants Y30, Y35 and unirrigated control (Z2), suggesting that MDA accumulation and related enzyme activities may be differentially regulated on the mutants X6 and Y20 compared to the other mutants^6^ or significant shortcomings emerge when it is used to assess lipid peroxidation on the related mutants^34^. The APX and GR are the key enzymes for ascorbate–glutathione (AsA–GSH) cycle and prevents the accumulation of a toxic level of H_2_O_2_ in photosynthetic organisms under stress conditions^35^. Although the APX and GR activities have been shown to increase under various stress conditions including drought in different plant species^32^, these enzyme activities were found significantly lower on the mutants than both control plants in this study, suggesting that oxidative damage did not occur at all on the mutants or had less deleterious effects in mutants compared to control plants.

The transcriptional and posttranscriptional levels of abiotic stress related genes in plants change under water deficit conditions^36^. The results of this study revealed that expression pattern of drought related *MtP5CS*, *MtDehyd*, *MseIF-2*, *MtRD2* and *MsNAC* genes differentially regulated on the mutants compared to control plants (Fig. 7). The presence of down regulation of all genes could be seen on the mutant X6 in both time intervals of drought stress (18d and 24d) while gene regulation pattern of the other mutants has changed based on the time and duration of drought stress (Fig. 7). The expression level of *MtP5CS* gene on the 18th day of drought stress were significantly lower in mutants than control Z2 with the exception of mutant X6 while prolonged drought stressed caused a down regulation of the same gene (Fig. 7). The expression levels of *MtDehyd*, *MseIF-2* and *MtRD2* genes on the 18th day of drought stress were upregulated on the mutants Y20, Y30 and Y35 although control Z2 showed a lower expression level than the mutants on the same time interval (Fig. 7). On the other hand, prolonged drought stress (24 d) caused severe down regulation of the *MtDehyd*, *MseIF-2* and *MtRD2* genes on the mutants although control Z2 had no or lower expression level than the mutants (Fig. 7). Both down and up regulations of the *MsNAC* gene expression were observed on the mutants at both time intervals (18 d and 24 d) while the same gene significantly up regulated on unirrigated control (Z2) plant at given time intervals. These results indicated that mutants have different mode of action in response to drought stress and transcriptional regulations of drought related genes tested in this study provided an early alert so that mutants become more tolerant against to prolonged drought stress in comparison to control. The results of drought related gene expressions also showed an agreement with the levels of enzyme activities determined on the mutants on the 18th day of drought stress (Fig. 7), except TBARS. Control plants showed higher SOD, APX and GR enzyme activities than the mutants on the 18th day of drought stress while the mutants Y20 and Y30 had lower levels of APX and GR enzymes on the 24th day of drought stress than control plants (Fig. 7). These findings suggested that transcriptional and posttranscriptional regulation of the mutants had unique mode of action in response to given drought stress conditions compared to the controls.

In conclusion, the results of this study revealed that screening of M_3_ alfalfa seeds in a root growth assay supplemented with 35% PEG_600_ was able to determine novel drought tolerant mutants which also tolerated water deficit conditions applied for 24 days after the first cutting at flower bud stage. However, the novel drought tolerant alfalfa genotypes determined in this study should be further evaluated under field conditions for hay yield, nutritional quality, nitrogen-fixing ability and sustainability.

## Materials and methods

### Material

We confirm that the experimental research and field studies on plants (either cultivated or mutants), including the collection of plant material were conducted based on complies with relevant institutional, national, and international guidelines and legislation. The 200 g seeds (thousand seeds weight is about 2 g) of alfalfa (*Medicago sativa* L.) cultivar Bilensoy-80 were mutagenized by using 0.15% ethyl methane sulfonate (EMS) for 12 h as indicated in the literature^37,38^. About 470 g M_2_ seeds were obtained from the field grown M_1_ plants. The M_2_ seeds were planted with 70 cm raw space. The M_2_ plants were isolated with isolation bag at early budding stage and have been left for selfing which was continued approximately one month during flowering periods. Pods of those plants were harvested by hand and were trashed manually. A total 340,675 M_3_ seeds were used for in vitro screening by using root growth assay given below.

## Methods

### Root growth media and planting M_3_ seeds under in vitro conditions

The M_3_ seeds were treated with pure ethanol for 10 min, then kept in a solution containing HCl (0.5 ml/100 ml) and HgCl_2_ (0.2 g/100 ml) for 20 min and then were washed with sterile dH_2_O for 5X. Half-strength MS medium containing 5 g/L sucrose, 1% agar and 2 mM MES buffer with pH 5.7 was poured into disposable (sterile, 3 × 15 cm) plastic petri dishes under aseptic conditions and were left to cool down. To induce drought stress, 35% PEG was added on the top of solidified half-strength MS medium by using the infiltration method (Fig. 1)^2,39^. The seeds of Bilensoy-80 cultivar were used as control (Fig. 1B). Petri dishes, taped with porous 3 M Micropore tape were kept at 4 °C for 48 h in order to break possible seed dormancy. Subsequently, the petri dishes were incubated in a constant dark condition for 3 days at 25 °C and for another 4 days at 25 °C in an upright position in a controlled plant growth chamber with a 12 h light (350 µmol m^−2^ s^−1^)/dark cycle^15,39^.

### Growth and screening of M_3_ mutants

Seeds germinated and showed a good root elongation were identified as drought tolerant candidate mutants (Fig. 1C,D) and were removed from petri dishes by using a forceps and were transferred to viols (5 × 5 cm) filled with peat-perlite mixture (3:1 v/v) (Fig. 1E). The viols were kept for 14 h of light (350 µmol m^−2^ s^−1^) until the first true clover leaves were visible (Fig. 1F) at 20 °C and 65% humidity growth conditions. The seedlings showing the first true leaves (three leaflets, 7–10 cm seedling height) were then transferred into the pots (30 cm × 30 cm) containing peat-perlite (3:1 v/v) mixture (Fig. 1G). Plants were grown till bud stage under given growth conditions as described before (Fig. 1H).

### Application of drought stress to M_3_ plants

The candidate mutants were re-evaluated under water deficit conditions applied for 24 days after the first cutting at bud stage. The M_3_ mutants were grown under given conditions as described before until the first flower buds of the main stem visible and were cut at a height of 5 cm (Fig. 1H). The pots were then irrigated until they reached the field water capacity and were left for 24 h to allow the water to drain^6,40^. No irrigation was applied to those pots for a total of 24 days and the leaf samples were taken at 0th (control, before cut), 18th and 24th of the drought stress, and were immediately stored at − 80 °C till used for physiological and molecular analysis. The agro-morphological parameters were also determined at given time intervals of drought stress and the results were compared with irrigated (Z1) and unirrigated (Z2) controls^6^.

### Determination of agro-morphological parameters of M_3_ plants under water Deficit conditions

Main stem length, main stem thickness, the number of branches, the number of leaves, middle leaflet length and width, and plant canopy temperatures were determined on the 18th and 24th day of drought stress. Five randomly chosen pods from each plant were used to determine seed yield per pod and the amount of M_4_ seeds obtained from each plant (g/plant) was determined. The main stem thickness was determined between the 2nd and 3rd branch of the main stem by measuring with a 0.1 mm dividing caliper. The length and width of the middle leaflets were determined from the 4th and 5th leaves of the main stem^6^. Before taking leaf samples, plant temperatures were determined using a laser-marked infrared thermometer (IR988) from 3 different points belonging to the lower, middle and upper parts of each plant.

### Determination of physiological parameters

The existing tissues on the 0th (control, before cut), 18th and 24th days of the stress were used to determine protein contents, superoxide dismutase (SOD) isoenzyme and thiobarbituric acid reactive substances (TBARS) levels, ascorbate peroxidase (APX) enzyme and glutathione reductase (GR) enzyme activities with three biological replications.

The bovine serum albumin (BSA) standard according to the Bradford method were used to determine total soluble protein content^41^. The content of thiobarbutyric acid (TBA)-MDA complex formed by this method was determined at A532 and A600 nm in the spectrophotometer. MDA contents in tissues were calculated using the following formula: MDA content = [(A532 − A600) × extract volume (ml)]/[155 mM/cm × sample amount (mg)]. The SOD enzyme activity was determined spectrophotometrically at 560 nm according to the method based on the photochemical reduction of nitrobluetetrazolium (NBT)^42^. The APX enzyme activity was determined based on given literature^43^. Total APX enzyme activity in tissues was calculated from the initial rate (nmol.ascorbate.min^−1^.mg protein^−1^) using the extinction coefficient of ascorbate (2.8 mM.cm^−1^). The GR enzyme activity was carried out as stated in the literature^44^. The total GR enzyme activity of the samples was calculated from the initial rate of the reaction (nmolNADPH.min^−1^.mg protein^−1^) after subtracting the non-enzymatic oxidation using the extinction coefficient of NADPH (6.2 mM cm^−1^).

### RNA isolation

Total RNA isolation of the leaf samples was completed by using the commercial RNA extraction kit (Vivantis GF-1) according to the protocol specified by the company. The quality of isolated RNAs were determined by spectroscopic measurement at 260/280 nm with a nanodrop device and were also confirmed on 2% agarose gel using a dedicated gel electrophoresis system to prevent different enzymatic contaminations (Supplemental Fig. 1).

### Determination of gene expression differences (RT-qPCR)

First-strand cDNAs were synthesized with 4 µl of total RNA RevertAid First-strand cDNA synthesis kit (Thermo Fisher Scientific, USA). Expression differences of specific genes for drought stress were detected by RT-qPCR (StepOne 7500, Applied bioscience) using the relevant gene-specific primers (Supplemental Table 1). The primers of all genes were tested to determine the optimal binding temperatures and amplicon status before RT-qPCR analysis. In addition, the specificities of the primers and PCR products were also tested and were confirmed by melt curve analyzes performed at the end of the RT-qPCR analysis. The RT-qPCR conditions were applied at 95 °C for 15 min, followed by 40 cycles of 95 °C for 40 s, 55 °C for 40 s, and 72 °C for 30 s. At the end of the PCR reaction, the melt curve analysis was performed at 58–92 °C. A dissociation kinetics analysis was done at the end of experiment to check the specificity of the annealing.

As housekeeping genes, *Ms18srRNA* and *MsActin* reference genes were simultaneously tested in the RT-qPCR analyzes^6,45^. Since the *Ms18srRNA* gene was found to be more stable and showed less variation between technical repeats, it was used as internal control in the RT-qPCR amplifications and the results were compared with the 2-delta-delta Ct method^46^. The MIQE guidelines were followed for all qPCR experiments^47^.

The gene expression levels were determined on the leaf tissues taken on the 0th (control, before cut), 18th and 24th days of drought stress with 3 technical replications. The settings recommended by the manual of the device (StepOne 7500, Applied bioscience) were used to determine the critical threshold values ​​(Ct) of the amplicons. The delta Ct standard deviation values among the technical replications ​​ ≥ 0.25 were repeated. In the calculations of the relative quantity (2-delta-delta Ct), the RQ (Relative Quantification) value in the control gene expression was accepted as 1, and more than 2 times or less than 0.5 times of the expression changes of the samples were used in the interpretation of the results. In addition, a logarithmic indicator chart was used in the creation of gene expression graphs, especially to see the changes in expression less than 0.5 times compared to the reference gene.

### Data analysis

The agro-morphological data (middle leaflet length and width, plant canopy temperatures and the number of seeds per pod) were subjected to one-way ANOVA using the SAS package program^48^. The physiological parameters were analyzed according to the methods in the relevant references specified in the method section. The differences between the means of the data were tested with the LSD test at the *P* < *0.05* level. Molecular data were normalized according to the 2-delta-delta Ct method using *Ms18srRNA* gene as internal control, and the expression levels of the relevant genes were determined^46,49^. The significant differences at *p* < 0.05 level were indicated with different letters above the columns of figures.

## Supplementary Information

Supplementary Information.
