# Supplementary material for: Improved drought tolerance of EMS mutagenized Alfalfa (Medicago sativa L.) mutants by in vitro screening at germination stage
Source: Sci Rep. 2022 Jul 26;12:12693. doi: 10.1038/s41598-022-16294-0 (PMC9325702; doi:10.1038/s41598-022-16294-0)
Supplement: Supplementary file 1 — Supplementary Information. [file 41598_2022_16294_MOESM1_ESM.pdf]

Supplemental Materials

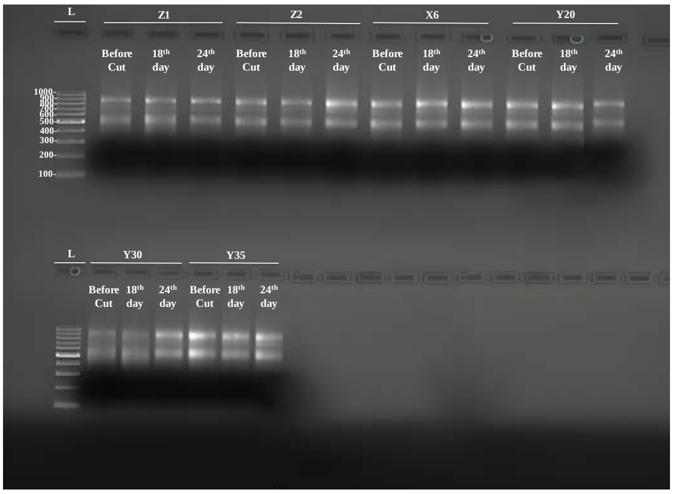

Supplementary Figure 1. Total RNA of samples. L, 100bp DNA ladder (Vivantis).

Supplementary Table 1. Gene-specific primers, their forward (F) and reverse (R) sequences used in RT-qPCR.

Gene	Primer sequences (5' to 3')	References
MtP5CS	F: TCAGAGGACTACGTGTTGGA<br>R: ATGAGTACTAAGCAGAGAGG	[1]
MtDehyd	F: GAGCGAGGAGGAAGTTGATGG<br>R: TGGTGCTGGTGGAGTTGTTA	[2]
MsEIF-2	F: GGTGCTGGGTCATCAAAGG<br>R: GCTCTGGGTCCTGGACAAC	[3]
MtRD2	F: GCAGCTGTGGTTCTGGGGACC<br>R: AGCAATACTCACCGACGCTTCCT	[2]
MsNAC	F: TGGCTTTAGATTTCATCCAACTG<br>R: AATACCATTCATTCTCCCCAAAC	[2]
Ms18srRNA	F: GGGCTCGAAGACGATCAG<br>R: AGCCTTGCGACCATACTCC	[3]
MsActin	F: TCCTGGGTGCTCTTCAGGAGCAA<br>R: TAGGGCTGTGTTTCCAAGT	[2]

1. Yamada, M. et al. Effects of free proline accumulation in petunias under drought stress. *J Exp Bot* **56**, 1975-1981 (2005).
2. Quan, W., Liu, X., Wang, H. & Chan, Z. Comparative physiological and transcriptional analyses of two contrasting drought tolerant alfalfa varieties. *Front Plant Sci* **6**, 1256 (2016).
3. Castonguay, Y., Michaud, J. & Dubé, M.-P. Reference genes for RT-qPCR analysis of environmentally and developmentally regulated gene expression in alfalfa. *American Journal of Plant Sciences* **6**, 132-143 (2015).
